# Supplementary material for: Are carbon emissions trading and green financial instruments synergistic? -Comprehensive quantitative research based on content analysis
Source: PLoS One. 2024 Mar 7;19(3):e0298601. doi: 10.1371/journal.pone.0298601 (PMC10919601; doi:10.1371/journal.pone.0298601)
Supplement: S1 Data — (ZIP) [file pone.0298601.s001.zip › Data/地级市Z_F工作报告.zip!/╣π╢1⁄2/╔╪╣╪╩╨/└┤╫╘╣1⁄2╓┌║┼í╛┬φ┐╦╩2╛▌═°í┐_2008.docx]

各位代表：

我代表市人民政府向大会作政府工作报告，请予审议，并请各位政协委员和其他列席人员提出意见。

2007年工作回顾

2007年，在省委、省政府和市委的正确领导下，在市人大及其常委会、市政协的监督支持下，市政府团结和依靠全市人民，坚持以邓小平理论和“三个代表”重要思想为指导，全面贯彻落实科学发展观，按照中共中央、国务院和省委、省政府的部署，认真落实市委的决策，致力于建设粤北经济强市、交通枢纽、区域中心、山水名城、和谐韶关，突出抓好工业、农业、交通、城建、旅游等重点工作，抢抓机遇，锐意进取，市十二届人大一次会议确定的2007年预期目标圆满完成，经济社会发展取得显著成效，为争当山区科学发展排头兵打下了良好基础。

一、经济保持又好又快发展，多项指标实现重大突破

经济综合实力迅速提升。据统计，全市实现生产总值482.5亿元，比上年增长14.9%，增幅超过全省平均水平。三次产业结构由2006年的15.8∶46.2∶38调整为15.4∶47.3∶37.3。人均生产总值超过2000美元，工业增加值、固定资产投资、年末金融机构各项贷款余额均突破200亿元，进出口总额突破10亿美元，接待游客突破850万人次，多项重要经济指标上新台阶，经济运行呈现改革开放以来最好发展态势。

工业经济效益大幅度提高，节能降耗取得新成绩。全市工业增加值206亿元，增长16.6%。优势企业和支柱产业进一步发展壮大。规模以上工业企业当年新增84户、总数达446户，完成增加值176.7亿元、增长17.5%，占全部工业增加值的85.8%；主营业务收入增长23.9%，综合经济效益指数创历史新高。圆满完成万元生产总值能耗和工业增加值电耗下降目标任务。

财税收入实现有效增长，城乡居民收入稳步增加。来源于韶关的财政总收入88.1亿元、增长32.1%。全市财政一般预算收入30亿元、增长31.8%，增幅居全省第四位。国税收入55.7亿元、增长25.9%；地税收入25.4亿元、增长41.7%，增幅排全省第一。市区居民人均可支配收入13545元、增长12.5%，农村居民人均纯收入4306元、增长8.3%。

农业生产加快发展，综合能力进一步提高。战胜了严重旱情，防控重大动物疫病成效显著。农业增加值增长6%，创15年来新高。粮食种植面积271.9万亩，创2003年以来新高，总产量再次突破百万吨大关。生猪饲养量、出栏量分别增长5%和6.1%。基本农田保护加强，改造中低产田5.4万亩，恢复和改善灌溉面积8.4万亩。新增无公害农产品基地4.1万亩、绿色食品基地5万亩。乳源建成“巴西果国家级农业标准化示范区”。翁源成为全国最大的国兰生产基地。

旅游产业快速发展，服务业全面繁荣。第三产业增长16.6%，发展全面加快。旅游总收入达40.6亿元，增长59.6%。城乡市场两旺，全市消费品零售总额196.6亿元，增长19.4%。商品房销售面积159万平方米、销售额32.3亿元，分别增长53.3%和95.9%。

外向型经济发展提速，对外贸易大幅增长。据海关统计，全市进出口总额12.9亿美元，增长38.4%。其中出口5.3亿美元，增长21.1%。全市实际利用外资1.5亿美元，增长25.7%。新批外商直接投资项目86个，合同利用外资5.1亿美元、增长57.8%。有14个项目投资额超过1000万美元。海关通关货运量417.4万吨，增长2.5倍。沃尔玛在市区设立分店，引进世界500强企业实现零的突破。香港上市公司建溢集团大陆总部落户沐溪工业园。成功举办韶关招商引资推介大会，当年签约、奠基、投产项目390个，总投资426亿元。

民营经济持续健康发展，全民创业开局良好。年末个体工商户6.9万户，从业人员14.8万人，分别增长17.8%和14.8%；私营企业5326户，从业人员4.6万人，分别增长9.5%和12%。新增规模以上民营工业企业46户。东阳光公司铝板带产品（HFF）成为我市首个中国名牌产品。民营经济完成固定资产投资76.8亿元，增长35.6%，民营经济增加值占全市生产总值的比重达44%。

固定资产投资实现新突破，发展后劲明显增强。全社会固定资产投资完成217.6亿元，增长28.6%。重点项目完成年度投资85.7亿元，完成年度计划106.9%。武广铁路客运专线韶关段建设加快，韶赣高速公路粤境段全线动工，省道S248线乐昌至犁市段改造竣工通车。建成500千伏曲江—鲤鱼江间隔工程和220千伏武江站扩建等输变电工程。旭日玩具城一期、韶关冶炼厂扩建项目投产。丹霞冶炼厂开工建设，进展加快。

二、城市创建工作取得新成绩，建设山水名城迈出新步伐

成功创建全国双拥模范城。深入开展双拥工作和军民共建活动，国防教育、国防动员和民兵预备役工作不断加强。全国双拥工作领导小组、民政部、解放军总政治部首次授予我市“全国双拥模范城”光荣称号，这是我市继“中国优秀旅游城市”之后的又一“国字号”名片。

创建国家卫生城市和国家园林城市取得重大进展。全面整治市区“六乱”、“五小”行业和建筑工地、集贸市场、小街小巷、城中村及城乡结合部环境卫生。花拉寨生活垃圾填埋场首期工程建成并投入使用。市区垃圾中转站改造全面完成。食品卫生、公共场所和生活饮用水卫生、传染病防治、病媒生物防制工作基本达标，创卫进入申报考核阶段。据省创卫考核组初步评估，市区大多数指标达到国家卫生城市标准。市区绿化力度加大，新建绿地374.9公顷；建成区绿地率35.61%，绿化覆盖率38.24%，人均公共绿地7.78平方米，均达到省园林城市标准。

芙蓉新城建设强力推进，市区改造全面加快。编制了《韶关市城市总体规划（2006—2020年）》、《市区综合交通规划》、《丹霞山风景名胜区总体规划》等重要规划。韶关市区规划总面积、建成区面积分别达到168平方公里和78.3平方公里。加快推进芙蓉新城建设，市政、路网项目前期工作进展顺利。市公安局指挥中心落成，韶关大道、京珠高速公路韶关互通立交等工程建设加快。城市基础设施不断完善。新铺设供水主管22.5公里，建成鹅坑桥、工业西路排水管网。韶关碧桂园一期等大型高档商住区相继落成，风度商业广场竣工投入使用，帽峰公园改造和东堤路拆迁完成，东岗岭、东河职工新村危旧直管公房改造取得阶段性成果。

创建森林生态市成效明显，生态环境保持优良。完成造林作业面积32.4万亩。年末林木绿化率、活立木蓄积量分别达到71.7 %和7199万立方米。市区第二污水处理厂一期工程和曲江、南雄、始兴污水处理厂投入运行。城镇生活污水集中处理能力达14万吨/日，每年可减排化学需氧量3000吨左右。市区生活污水处理率达60%以上，空气质量达到国家优良标准。全市江河水质良好。

三、县域经济快速发展，新农村建设稳步推进

进一步健全县域发展激励机制，采取有力措施，大力推进工业化、城镇化、农业产业化进程，县域经济发展加快。县域生产总值、工业增加值、财政一般预算收入达到209.1亿元、75.4亿元和14.8亿元，分别增长17.3%、26.4%和34.5%，均高于全市平均增长水平。地方财政一般预算收入曲江区突破3亿元，乳源瑶族自治县突破2亿元，武江区、浈江区均突破1亿元。有5个县（市）工业增加值超过农业增加值。支持县域交通、能源、水利、工业园区等基础设施建设。各县（市、区）均与珠三角签订联手推进产业转移合作协议，工业园区基础设施进一步完善，承接产业转移能力明显增强。

农业农村投入明显增加，新农村建设扎实推进。支农惠农政策进一步落实，农村固定资产投资达32.2亿元、增长29.7%。完成农村公路路面硬化850公里。解决了69个无电村（村民小组）的用电问题。加快水利防灾减灾和小型水库除险加固工程建设，初步清理整顿“四无”小水电。加强气象保障服务，减灾增效明显。全面开展省级村庄整治规划试点村规划编制工作。农村“一池五改”扎实推进，农村生产生活环境进一步改善。帮助1.37万农户完成住房改造，农村危房改造任务基本完成。新建4万个沼气池。全市已有60%的农户住上楼房或红砖瓦房，13.1万农户用上沼气，95%的行政村实现通水泥公路。新农村创业人才培训、农村劳动力和未转移人员培训共2.9万人。农村信息惠农工程成效显著。全市药品供应网乡镇覆盖率达98.9%、行政村覆盖率达94.8%，药品监督网实现全覆盖。全市行政村卫生站基本落实了“一村一站一名乡医一万元”的政策。96%的农业人口参加了2008年度新型农村合作医疗。

四、社会事业全面发展，多个领域继续保持先进水平

实施科教兴市战略取得新成绩。重大科技攻关、公共技术服务平台建设取得新突破，粤港关键领域科技招标项目中标3项、居全省山区市之首，建成了液压油缸特色产业基地数字化检测平台和行业专利数据库，包括中美日等七国和两个国际组织的集成专利数据库落户我市。发明专利申请量连续3年居全省山区市之首。科技创新结硕果，荣获国家科技进步二等奖1项、省科技进步奖7项。新增4家高新技术企业、5家省级民营科技企业、1家省级工程中心。科技企业孵化基地首批项目成功进驻。教育事业发展加快，教师队伍建设进一步加强，教育质量和办学水平稳步提高。青少年信息学竞赛成绩连续十年居全省第一。全面完成农村中小学义务教育学校C、D级危房改造。全市户籍人口高中阶段教育毛入学率达80%，居东西两翼和粤北山区之首，比全省平均水平高近15个百分点。每万常住人口高中阶段在校生495人，为全省平均水平的153%。每万人口高考本科上线人数比例居全省第七位。北江中学、翁源中学、南雄中学成为国家级示范性普通高中。推进职业技术教育区域中心建设，高效率建成市技工教育及农民工培训示范基地，省高技能人才公共实训基地落户我市。中等职业教育每万人口在校生居全省前列，其中技工教育在校生占全省十分之一强，成为我省技工教育大市。高校办学水平稳步提高，全日制在校生总数达2.3万人，居全省第5位。

医疗卫生和计划生育等事业有新发展。公共卫生水平居全省前列，连续四年没有发生甲、乙类传染病暴发疫情和重大食物中毒事件。市直公立医院医疗质量和服务水平明显提高，人均门诊、住院费用连续四年在全省各市处于最低水平。建设中医药强市工作扎实推进，3间中医院、3个专科分别被列入省中医名院和国家中医特色专科建设项目。市卫生局荣获“全国卫生系统先进集体”称号，是我省30年来唯一获此殊荣的卫生行政部门。完成省下达的人口计划。当年出生人口计划生育率达92.1%、创历史新高，全市人口性别比同比下降3.99个百分点。文化体育事业有新进步。有17件舞台作品获省以上各项奖励，5件作品获省“五个一工程”奖，《囚犯生死大转移》获中国纪录片国际选片会“十佳纪录片”大奖。地方志编纂工作走在全省前列。我市运动员代表省参加第七届全国残疾人运动会夺得“两金两银”，参加第八届全国少数民族传统体育运动会夺得“四金十一银”。民族地区经济社会事业协调发展。宗教领域和谐稳定。外事、侨务、统计、人防、地震、档案、水文监测等工作取得新成绩。

思想道德建设深入推进。广泛开展“爱国、守法、诚信、知礼”现代公民教育、社会主义荣辱观教育和精神文明创建活动，涌现出大批文明单位、文明村镇、文明社区和文明家庭，涌现出农民致富带头人邓素梅和“感动韶关十佳道德模范”邓玉莲等一批先进典型。认真实施妇女儿童发展规划。加强未成年人保护和思想道德建设。大力推进“六好”平安和谐社区建设和“百镇千村”平安工程，24个社区被评为广东省“六好”平安和谐社区。

五、重视解决民生问题，和谐韶关建设成效明显

坚持以人为本，认真解决人民群众最关心、最直接、最现实的利益问题。大力促进就业。新增就业岗位5.3万个，下岗失业人员再就业2.3万人，其中就业困难对象再就业4117人，市区实现无“零就业家庭”。城镇登记失业率为3.15%。农村劳动力转移就业6.3万人。社会保险覆盖面扩大，追缴历史欠费1.4亿元，社保基金平稳运行。落实养老保险新政策，筹集2.4亿元完成养老保险上调目标，人均上调240元。在全省率先启动城镇居民基本医疗保险试点和被征地农民养老保险试点工作。清理历史拖欠农民工工资3000多万元，劳动关系进一步和谐稳定。市劳动保障局被评为全国劳动保障系统优质服务窗口和国家首批金保工程建设示范单位。城乡居民最低生活保障和社会救助工作进一步加强。全年新增低保对象8843人，低保和五保对象实现应保尽保。免收了农村义务教育阶段学生课本费，对7.4万名困难家庭学生住校寄宿实行生活补助。对市辖三区低保、零就业和残疾人家庭子女从小学至高中阶段教育实行了免费入学。发放基本医疗救助金370多万元，救助1.7万多人次。救助各类流浪乞讨人员6142人次。新建城镇廉租房和经济适用住房17.8万平方米，城镇低收入家庭住房保障水平明显提高。市建设局被评为全国建设系统先进单位。扶贫工作进一步加强，年纯收入1500元以下的贫困人员比上年减少8.7万人。去年承办的八件实事，除望韶楼正在施工、今年可竣工投入使用外，其余七件均已完成。

加强公共管理，经济社会秩序良好。加强国土资源管理，严格保护耕地，依法征用土地，加强矿山整治。严格依法治价管费，加强产品质量和食品药品安全监管。完善打击传销活动综合治理机制，始终保持高压态势打击传销活动，遏制了传销活动蔓延势头。整治虚假违法广告，有效保障市场供应和消费安全。落实安全生产行政首长负责制，深入开展安全生产综合建设试点工作，有效防范重特大安全事故发生，安全生产形势持续向好。坚持打防管控并举，大力整治重点治安问题，严重刑事犯罪明显减少，命案破案率居全省前列。建立健全人民调解网络，认真处理群众来信来访，大力开展社会治安和涉法涉诉信访问题专项治理，扎实做好矛盾纠纷排查调处工作，有效化解人民内部矛盾，维护了社会和谐稳定，我市连续8年被评为全省社会治安综合治理先进市。

六、加强政府自身建设，行政效率明显提高

民主法制建设进一步加强。认真执行市人大及其常委会决议并做好工作报告，坚持向政协通报工作情况。办理人大议案1件、代表建议131件、政协建议案4件和委员提案130件，代表、委员满意率均达99%。密切与工会、共青团、妇联等群众团体的联系，认真听取社会各界意见。村（居）民自治制度不断健全。推进依法治市，开展“五五普法”教育。坚持依法行政，加强行政复议、行政监察和执法督查，建成行政许可审批电子监察系统。全面贯彻实施《公务员法》，公务员队伍建设进一步加强。完成第三批行政审批事项清理工作，确定保留行政许可95项，削减91项，减幅达48.9%。清理规范性文件327项，修改48项，废止153项。加强制度建设，颁布规范性文件7件。坚持重大事项集体决策、专家咨询论证和决策听证、公示等制度，聘请了28名市政府顾问，政府科学决策、民主决策、依法决策水平不断提高。

开展机关“效率年”活动成效明显。机关工作人员事业心和责任感进一步增强，办事效率和服务质量全面提高，政府社会管理和公共服务职能明显增强。办好“市长信箱”和“民声热线”，政群关系进一步密切。市行政服务中心实行“一站式”办公和联办代办服务制度，初步建成“行政便民超市”。

认真落实中共中央关于党风廉政建设和反腐败斗争的部署和要求，健全教育、制度、监督并重的惩治和预防腐败体系。强化党风廉政建设责任制，切实加强领导干部廉洁自律教育和干部队伍建设。严格禁止公务人员利用职务便利谋取不正当利益，加强对财政预决算、专项资金使用和领导干部经济责任审计，推进政务、厂务、村务和事务公开，大力纠正损害群众利益的不正之风，强化对权力运行的监督。

各位代表，2007年是我市经济社会发展取得明显进步的一年，全市人民迎来了加快发展的新局面。这是全市人民团结协作、心齐实干、开拓创新、艰苦努力的结果。在此，我代表市人民政府，向全市广大工人、农民、知识分子、干部职工，向驻韶人民解放军、武警部队官兵、人民警察和各民主党派、人民团体、社会各界人士，致以崇高的敬意！向长期关心支持韶关发展的港澳台同胞、海外侨胞、驻韶中省单位干部职工和国内外朋友，表示衷心的感谢！

在看到成绩的同时，我们也清醒地认识到，工作中还有许多不足之处，经济社会发展面临不少困难和问题。主要是：全市经济总量仍然偏小，县域经济发展不平衡并且水平仍然较低；改革创新的力度不够大，体制改革的任务仍然艰巨；部分企业职工工资增长不快，农民收入实现较快增长仍很困难；食品类价格上涨幅度较大，城乡低收入居民生活比较困难；政府职能转变没有完全到位，公共管理和服务水平还需进一步提高。对此，我们将以对人民高度负责的精神，认真研究并采取有力措施加以解决。

2008年工作安排

2008年是全面贯彻党的十七大精神、实施“十一五”规划的关键之年。我们必须乘势而上，继续解放思想，坚持改革开放，推动科学发展，促进社会和谐，认真贯彻党的十七大和省委十届二次全会、省十一届人大一次会议及市委十届四次全会精神，更加注重改善民生，更加注重城乡、区域协调发展，扎实开展“节能减排、防灾减灾、扶贫帮困、就业再就业、群众安居、教育普及与提高、城乡医疗、文化惠民、创业人才培训、可持续发展”等十大民心工程，推动经济社会全面转入科学发展轨道，争当全省山区科学发展排头兵。

2008年全市经济社会发展主要预期目标是：生产总值增长13%左右，单位生产总值能耗下降不低于4.5%，完成省下达的节能减排任务，全社会固定资产投资增长20%，社会消费品零售总额增长16%，实际利用外资增长18%，外贸进出口增长15%，地方财政收入与经济同步增长，居民消费价格涨幅控制在4%以内，城镇居民登记失业率控制在3.5%以内，农村居民人均纯收入增长7%，市区居民人均可支配收入增长8%，人口自然增长率控制在7.18‰以内。

重点推进八个方面工作：

一、继续解放思想，坚持改革创新，为科学发展提供强大动力

我们要围绕科学发展这个主题，推动新一轮思想大解放。通过解放思想，更新观念，以世界眼光来谋划韶关发展大计，以战略思维来寻求破解发展难题、应对挑战的新办法新举措。当前，要树立三种意识：一是树立开放意识。以更加宽广的视野去谋划韶关的发展，以更加奋发有为的精神状态去迎接挑战，以更加博大的胸怀去创造宽松的发展环境，吸引更多的资金、人才、技术流向韶关。二是树立机遇意识。要加强学习，全面提高把握机遇的能力。三是树立创新意识。要敢于创新、善于创新、勇于创新，通过创新促进大发展。

今年是全市“解放思想深化改革年”。我们要紧紧围绕争当实践科学发展观排头兵这一总要求，增强责任感和使命感，以新一轮改革开放推动新一轮大发展。一是深化以产权制度为核心的国有企业改革。推进国有及国有控股公司产权多元化。依托市属优势企业或通过引进战略投资者，加快优势产业振兴步伐。进一步完善公司法人治理结构，规范经营管理者的权利和责任，建立健全考核分配、经营预算和财务监管制度。二是深化投融资体制改革。引导各类资本投向生产、基建领域，实现投资主体多元化。加快现代市场体系建设，积极培育资本、劳动力、技术、信息等要素市场，促进要素资源向各个经济领域优化配置。规范实施企业投资项目备案制，健全政府投资项目决策机制，完善代建制。三是深化行政管理体制改革。健全政府职责体系和公共财政体系。简化行政审批程序，强化社会管理和公共服务职能。完善行政执法体制，规范执法行为。坚持依法治税，加强税收征管，提高纳税服务水平，建立和谐征纳关系。继续完善收支两条线管理。深化部门预算改革，提高财政支出绩效。严格执行预算，从严控制追加支出。加强支出管理，努力节约各项开支，确保重点支出需要。四是深化农村综合改革。稳定农村土地承包制度，引导土地承包经营权有序流转。大力扶持林业灾后复产，稳步推进集体林权制度改革，发展林业产业，着力营造林业发展的良好环境。抓好消雪岭华侨农场的改革改制。五是深化社会事业改革。坚持分类改革和管办分离的原则，加快事业单位改革，稳步推进人员聘用制度建设。在继续加大政府对公共服务和公共产品投入的同时，吸引各类资本参与社会事业的改革和发展，努力构建公平高效的社会事业服务体系。

推进和落实符合科学发展观要求的经济社会发展水平综合评价工作。建立科学的评价制度，制定实施方案，定期进行定量评估，全面分析经济社会发展水平状况和问题，为经济社会发展提供科学的决策依据。

二、承接产业转移，推动全民创业，走新型工业化道路

继续抢抓国际国内产业转移的良好机遇，加快外经贸发展。着力提高招商引资质量，加大对重大项目的招商力度，力争引进一批产业链长、科技含量高、创税能力强的龙头项目，在引进大企业、大项目上取得更大突破。加快对接广州、融入珠三角步伐，主动承接产业转移，在抓好外源型经济的同时，积极推进内联招商，重视引进民营制造业项目。加强对外招商策划推介，加强对台、对日韩招商工作，精心组织各项重大经贸活动。抓好2007韶关招商引资推介大会等重大经贸活动成果的落实，提高项目履约率和成功率。落实重大项目综合协调和跟踪服务机制，提高服务水平，努力营造一流的营商环境。完善招商引资责任制，抓好专业招商队伍建设。强力推进产业转移工业园建设，完善园区招商的体制、机制，加快项目入园步伐。大力促进加工贸易转型升级，鼓励和引导外资企业扩大内销。优化进出口产品结构，推动对外贸易稳步发展。同时，进一步完善口岸基础设施，营造低成本、高时效的通关物流环境。加快推进“内陆港”项目建设，规划建设韶关保税物流中心。实施现代物流发展规划，加快曲江、龙归、浈江等物流园区规划建设，扶持优势物流企业发展壮大，建设粤北粮油物流交易中心，把物流业培育成为新的支柱产业，加快区域物流中心建设。

加快推进全民创业。认真落实鼓励全民创业、发展民营经济的各项政策措施，加强对各类创业主体的引导、扶持和服务。放宽准入条件，降低创业门槛，规范收费行为，严禁以罚代管，有效减轻创业、发展成本。安排全民创业发展资金，实施创业富民培训工程和高技能人才培训工程，健全全民创业服务体系。建立全民创业项目库，加快小企业创业园区建设。积极引进创业投资基金和风险投资基金，做好发展利用资本市场工作，力争一批优势民营公司改制上市。

强力推进新型工业化，加快打造新兴制造业基地。抓住我省建设现代产业体系、加速发展重化工业的新机遇，支持优势骨干企业做大做强，全面提升钢铁、铅锌、机械装备与零配件、电力、烟草、生物制药、玩具等优势产业发展水平。实施产业集群发展专项规划，积极培育发展冶金、机械装备、玩具三大产业集群。推进全国最大的铅锌生产基地建设，延伸有色金属产业链。发展汽车零部件、建筑成套机械、机电设备工业，建设液压油缸、铝箔、有色金属材料特色产业基地，并力争装备制造企业对接、配套广州汽车产业有新突破。发挥粤北玩具检测中心作用，支持玩具企业发展，培育、引进关联企业，提高玩具产业集聚水平。加快自主创新步伐，推进以企业为主体、产学研相结合的产业技术战略联盟建设。做好知识产权、技术标准和科技领军人才工作，加强科技企业孵化器基地建设，打造粤北科技成果产业化中心。加快建设和完善产业公共技术服务平台。坚持实施名牌带动战略，着力引导名牌产品做强做大，形成若干拳头产品。

三、统筹城乡发展，壮大县域经济，加快新农村建设步伐

认真落实统筹城乡协调发展方略和“多予、少取、放活”的方针，以建立农民增收长效机制为核心，以农业农村经济结构战略性调整为主线，以农业综合生产能力建设为重点，以科技进步为先导，以深化农村改革为动力，以提高农民素质为基础，加强统筹协调，搞好分类指导，整体推进农村工业化、城镇化和农业产业化，大力促进农村经济繁荣和社会进步，加快农村全面建设小康社会步伐。

加快发展县域经济。支持各县（市、区）走特色产业发展之路，以工业化推动城镇化和农业产业化。着力支持各县（市、区）建设产业转移工业园和特色产业项目，集约发展园区经济。科学规划产业布局，强化城乡规划管理，配合省做好主体功能区规划编制工作，优化我市区域发展布局。加大县域交通、能源、水利、供电等基础设施建设力度，加强各县（市）城区、省级中心镇和专业镇建设，增强城镇聚集生产要素能力。继续完善和实施县域经济考核办法和激励型财政政策，充分调动市直单位和县（市、区）、镇、村的积极性，增强县域经济发展内在动力。

大力发展现代农业，加快打造农产品生产加工基地。转变农业发展方式，提高农业市场化、专业化、规模化水平。加快农业科技创新、科技普及和农业先进适用技术推广。加强动植物疫病防控，推进农业标准化建设，保证农产品质量安全。完善以农业技术推广、信息服务、农产品质量安全检测和农村金融服务为重点的农业社会化服务体系。鼓励创建农业品牌，推动标准化生产和农产品质量认证。发展壮大农业龙头企业，扶持发展农民专业合作组织。优化农业结构和农业生产布局，围绕七大传统主导产业和茶叶、药材、食用菌、花卉、糖蔗、蚕桑等特色产业，发展优质农产品生产，重点抓好百万亩优质蔬菜、百万亩优质稻、百万亩生物能源、300万头生猪、300万亩竹子等五大农业产业基地建设。发展农产品加工业，建设农业和农产品加工流通专业镇，推动“一镇一品、一村一品”发展。落实粮食工作责任制，完成省下达的粮食储备和粮食风险基金规模任务，确保我市粮食安全和总量平衡。

落实强农惠农政策，加大各级财政支农投入，引导更多的信贷资金和社会资金投向农业农村。加强水利、交通、电力、气象等防灾减灾工程及农村环境保护和环境卫生建设。做好灾后复产工作，严格保护耕地，加强基本农田建设，提高农业综合生产能力和抵御自然风险能力。完成600公里镇通行政村公路硬底化。加强村庄规划、建设和管理，建好80个省级试点村。制定和实施农村泥砖房改造工程规划。力争65%的农户住上钢筋水泥楼房或红砖瓦房，35%以上的农户用上沼气。新建一批农村学校大型沼气池集成技术示范点，扩大实施石灰岩地区节水技术示范工程。加快实施科技信息惠农工程，促进科技知识、市场信息进村入户。继续加强扶贫开发。推进农村集体经济组织化管理。加快农村社会事业发展，广泛开展文明、和谐、平安村镇创建活动，创建一批省级卫生村镇、生态文明村镇，提高农村文明水平。

四、推进旅游旺市，建设山水名城，打造一流生态环境

强力推进旅游产业化，着力打造旅游休闲基地。抓好“大丹霞、大南华、大南岭”精品景区和温泉度假基地的规划建设，构筑“大环境、大市场、大项目、大产业”发展格局。启动创建中国最佳旅游城市工作，支持仁化、乳源创建中国旅游强县。实施丹霞山总体规划和大南华地区综合开发规划。推进丹霞山申报世界自然遗产，创建国家5A级旅游景区和全国文明景区。加大旅游招商力度，推进旅游景区深度开发，创建更多国家4A级旅游景区。加快旅游基础设施建设，完善旅游道路和其他配套设施，实现各景区间快线连接。建设韶关旅游咨询服务中心。深化旅游体制改革，完善旅游政策、产品、营销和人才支撑体系。推进旅游与文化的结合。做好假日旅游工作。加快旅游业对接广州、融入珠三角步伐，推进与主要客源地战略投资者、旅游集团的合作。创新旅游推介营销方式，包装和推介一批精品项目，积极开发北京、上海等新客源市场。优化消费环境，巩固提升批发零售、餐饮住宿、交通运输等传统服务业，加快发展信息、商务、金融、物业管理、社区服务等现代服务业，引导房地产业健康发展，推动消费结构升级。

加大创卫创园工作力度，进一步优化城市人居环境。全面完成各项创卫整改任务，努力达到申报考核的必备条件，争取创卫成功。认真实施《韶关市城市绿地系统规划》，严格实行绿色图章审批制度。按照凸显自然山水、传承历史文化、抓好人工绿化的要求，大力实施创园增绿工程，做好建绿、透绿、复绿工作，争取创建省园林城市取得成功，确保创建国家园林城市工作取得重大进展。

做好市区控制性规划、给排水规划以及公共交通枢纽和对外联系主要干道定线规划等编制工作，推动城市建设纳入科学布局轨道。加快芙蓉新城道路、供水、供电、通讯等设施建设，确保韶关大道、京珠高速公路韶关互通立交项目按期建成通车。全面落实《芙蓉新城征地拆迁一揽子方案》，启动芙蓉新城征地拆迁安置新农村建设工程，妥善解决失地农民生活出路问题，完成征地拆迁任务。抓紧完成芙蓉新城相关地块和新韶关站广场及周边的控制性详细规划编制，确保韶关新火车站建设顺利推进。

认真抓好土地管理、耕地保护、节能减排等目标责任制的落实。一是推进节约集约用地。严格执行土地利用年度计划。抓好土地利用总体规划修编，优化土地布局和结构，加快盘活闲置土地，推进旧村、旧区、旧厂整理，提高现有用地使用效益，确保重点项目和产业建设用地需求。二是加强节能减排。加快实施节能减排工作方案和计划，把污染减排任务完成情况纳入环境保护责任考核。做好第一次全国污染源普查。严格限制高耗能、高耗水、高污染产业发展，确保按计划、按要求完成淘汰落后生产能力任务。防止低水平重复建设。鼓励发展能源资源消耗低、附加值高的产业。加强项目建设的环境评价和监督管理。加大环保执法力度，抓好重点行业、重点企业的治污减排、节能降耗工作。推进韶钢节能减排工程和粤北危险废物处理处置中心建设，开工建设仁化、翁源、乳源生活污水处理厂。抓好建筑节能工作，大力推广使用新型墙材。三是发展绿色经济。培育绿色产业，构建节约型产业体系。开展循环经济试点工作，创建一批清洁生产示范企业。抓好绿色建筑工程试点示范工作。加强自然保护区、风景名胜区、水源保护区、生态公益林区保护。实施城市生态文明亮点工程，抓好北（东）江林分改造工程和绿色通道、城区出口造林工作，完善林业灾害防御体系，治理水土流失。

五、努力扩大投资，加强重点建设

拓宽投资来源渠道。转变财政支持经济发展方式，综合利用国债、税收、贴息、担保等扶持手段，不断拓宽投入领域，增强财政调控能力。加强社会信用体系建设，进一步改善金融生态环境。完善金融服务平台，创新金融产品和服务，改进金融工具和技术，加强政银企合作，促进金融产业加快发展。加强和改进金融监管，优化信贷结构，防范金融风险，切实维护金融债权。继续做好农信社改革和新型农村金融机构试点工作。积极落实利用国家开发银行贷款资金，用好授信贷款额度。积极争取中央和省的各类政府性投资，扩大投资规模，增强经济发展后劲。

抓好重点项目建设。2008年计划安排续建和新开工重点建设项目39个，年度投资120亿元。加快综合运输、能源、防灾减灾、高新技术、加工制造、城乡建设、旅游休闲、民生保障、现代物流、生态环保工程等项目建设。支持东阳光化工基地、丹霞冶炼厂、坪石B电厂三期等项目加快建设，力争乐昌峡水利枢纽工程、湾头水利枢纽工程、韶关发电厂2×60万千瓦机组等项目开工建设。做好20个重点预备项目的前期工作，力争更多的预备项目转为开工项目。

推进交通枢纽城市建设。大力推进武广铁路客运专线韶关段、曲江至南雄高速公路等在建项目建设，争取开工建设韶关至赣州铁路广东段，加快广州至乐昌高速公路韶关段、大庆至广州高速公路新丰段前期工作。建设韶关港大坑口港区，整治北江中游航道韶关至大坑口段，加快改善航运条件。进一步提高城乡公交发展水平。

六、发展社会事业，促进文明进步

巩固全社会团结奋斗的思想基础。广泛开展社会主义荣辱观学习实践活动，深入开展“爱国、守法、诚信、知礼”公民道德教育和群众性精神文明创建活动，在全社会形成知荣辱、讲正气、守法纪、重诚信、促和谐的社会主义新风尚。切实维护妇女儿童的合法权益，加强青少年思想道德建设，促进青少年健康成长。加强科普宣传，倡导科学、健康、文明的生活方式。发展社会志愿者事业，引导和支持民间组织参与社会管理。加快民族地区经济发展，大力支持乳源瑶族自治县创建全国一流少数民族自治县工作。依法加强宗教事务管理，推动宗教界加大服务社会力度。加强国际合作交流，积极推进缔结国际友好城市工作。做好优抚安置工作，加强国防动员、国防教育、民兵预备役和人民防空工作，进一步发展新型军政军民关系，为争创“全国双拥模范城”二连冠打好基础。

优先发展教育，推进人才强市战略。继续巩固和提高义务教育发展水平，加强教师队伍和义务教育阶段学校规范化建设。做好城市免收义务教育阶段学生学杂费和课本费工作，实行城乡免费义务教育，进一步提高高中阶段教育毛入学率。重视加强农村师资力量，加大农村教育投入。推进城乡义务教育均衡发展。加快普及高中阶段教育。市一中新校区实现秋季招生。抓好市中等职业技术学校新校区和高技能人才公共实训基地建设，精心打造职业技术教育区域中心。建设高水平应用型地方大专院校，推动高等教育新发展。加强学前教育、特殊教育和民族教育。加快人才市场建设，做好招才引智工作，建设高素质高水平的专业技术人才队伍。

加快医药卫生事业发展，提高全民健康水平。推进医疗服务区域中心建设，加快建设覆盖城乡居民的公共卫生服务、医疗服务、药品供应保障和城镇医疗保障体系。提高疾病预防控制能力和突发公共卫生事件的处置能力，确保食品安全和公共卫生安全。加强对药品、保健品、化妆品和医疗器械的生产、流通和使用环节的监管，确保质量安全。新建粤北人民医院门诊大楼。加强农村三级医疗网络和城市社区卫生网络建设。创建更多的无政策外多孩出生镇（街）和无政策外出生村（居），加快后进地区的转化步伐，促进人口计生工作稳定、和谐发展。完善公共体育设施，实施全民健身和奥运争光计划，举办大型体育活动，丰富群众体育生活。认真抓好竞技体育业余训练工作，积极备战第十三届省运会。

加强社会主义文化建设。大力扶持公益性文化事业发展，稳步推进经营性文化单位改革，发展壮大文化产业。创新艺术院团发展机制，鼓励社会力量参与文化市场开发建设，扩大文化领域对外开放，不断增强文化发展活力。实施“文化惠民”工程，加强农村和城市社区文化设施建设，加快建设文化信息资源共享工程，构建公共文化服务体系。办好我市第五届客家山歌擂台赛、第六届新歌手大赛和第三届社区集体舞大赛，丰富群众文化生活。完善广播电视公共服务体系。发展韶关特色文化，促进文化与经济的结合。实施文化精品战略，繁荣文艺创作，推出一批体现时代精神、具有韶关特色、在全国有影响的文化精品。做好第二届《韶关市志》的总纂和出版发行工作。加强文物和非物质文化遗产的保护和利用，做好第三次全国文物普查工作。建设北伐纪念馆。办好张九龄诞辰1330周年纪念活动。

进一步加强统计基础建设，认真做好第二次全国经济普查工作。做好外事侨务、档案、气象、地震、水文监测等各项工作。

七、高度关注民生，维护社会和谐

完善覆盖城乡的社会保障体系，提高社会保障水平。积极探索统筹城乡社会保障新机制，加快推进社会保险体系建设。贯彻落实《劳动合同法》，加大参保扩面工作力度。扩大被征地农民养老保险和城镇居民医疗保险试点，提高新型农村合作医疗保障水平。调整提高企业退休人员基本养老金。完善社会保险市级统筹制度，确保社保基金安全运行。贯彻落实《就业促进法》，实行积极的就业政策。力争新增城镇就业岗位5万个，实现城镇下岗失业人员再就业1.2万人、农村富余劳动力转移就业4.5万人。实施充分就业社区（村镇）工程，发展就业容量大的产业和企业。完善县、镇、村三级劳动力公共就业服务机构，加强就业服务网络建设，加强创业和就业培训，以培训促进就业，以创业带动就业。加强再就业援助和就业引导，重点解决“4050”人员、残疾人等困难群体的就业问题。做好高校、技校、职校毕业生就业工作。加强劳动保障执法维权，推进劳动关系和谐。

加强帮扶困难群众工作。确保广大低保户、五保户的基本生活，建立低保、五保对象大病救助基金，加强医疗救助工作。加快完善住房保障体制，努力解决困难群众住房问题。落实高中阶段教育和高等教育学校助学制度，努力解决贫困学生“读书难”问题。加强法律援助工作。积极发展妇女儿童、老年人、残疾人福利事业和慈善事业，拓宽救济渠道，做好社会救助工作。突出为民办好八件实事：1、全面实行城乡免费义务教育。2、兴建韶关市职业病防治中心。3、提高城乡最低生活保障标准和五保供养标准。4、改造东河职工新村等危旧公房。5、动工兴建一批廉租房。6、免费开放韶关国家森林公园。7、建设市中等职业技术学校新校区。8、兴建韶关市五里亭消防站。

扎实推进“平安韶关”建设，促进社会和谐。坚持安全发展。进一步落实安全生产责任制和消防安全责任制，加强对高危行业、非煤矿山和公共场所的安全监督、隐患排查和专项整治，防范重特大事故。加强和改进社会管理。完善应急管理机制，健全应急体系，提高应对突发公共事件的能力。整治和规范市场经济秩序，落实价格调控政策，强化市场价格监管，增强调控市场的能力，保障市场有效供给，保持价格总水平基本稳定。完善公安机关指挥中心平台和治安监控网络，健全社会治安防控体系，落实社会治安综合治理各项措施，着力整治突出治安问题，严厉打击严重刑事犯罪。继续推进“六好”平安和谐社区建设。做好群众来信来访和人民调解工作，及时调处和化解各种社会矛盾纠纷，依法妥善处置群体性事件，维护社会稳定。

八、加强自身建设，提高行政效能

把遵守宪法和法律作为施政的根本原则。认真落实人大及其常委会各项决议决定，依法向人大及其常委会报告工作。主动加强与人民政协的联系，并向其通报工作，支持政协委员履行职责。认真接受人大及其常委会的监督、人民政协的民主监督和新闻舆论监督、人民群众的社会监督。认真实施人大议案，重视办好人大代表、政协建议案和政协委员提案。广泛听取各民主党派、工商联和无党派人士的意见，支持工会、共青团和妇联等人民团体开展工作。深入推进“五五普法”。做好市第四届村委会和第三届社区居委会换届选举工作，巩固和扩大基层民主。严格按照法律授予的权限管理经济社会事务，增强政府依法行政能力。加强行政执法督查，落实行政执法责任制、过错责任追究制和重大行政处罚备案及审查制度，做到有权必有责、用权受监督、侵权须赔偿、违法必追究。加强学习型政府建设，提高行政决策水平。完善重大决策专家咨询、社会公示、社会听证和责任追究制度，推进政府决策的民主化和科学化。

加快转变政府职能，提高行政效能。巩固提高机关“效率年”活动成果。建立健全领导干部落实科学发展观的政绩考核评价、县域经济考核、国有企业经营者考核、机关绩效考核、重点建设项目考核和信访维稳考核等制度，以落实制度推进责、权、利相统一。继续清理行政审批事项，减少会议文件。进一步完善行政服务中心“一站式”办公和联办、代办服务机制，推进市县两级服务体系建设。规范行政审批，简化审批程序，提高服务质量。

推进廉政建设上新水平。强化行政监察，严格执行党风廉政建设各项规定，加强执法专项监察，保证政令畅通，严肃查处政府工作人员违法违纪案件，健全机关效能投诉机制，落实行政首长问责制。坚决纠正和制止损害群众利益、扰乱市场秩序、破坏生态环境的行为，开展民主评议政风行风，构建治理商业贿赂的长效机制。坚持新时期党的群众路线，进一步扩大政务、厂务、村务和事务公开，保障人民群众的知情权、参与权、表达权和监督权。畅通社情民意反映渠道，坚持办好“市长信箱”和“民声热线”。强化审计监督工作。加快惩治和预防腐败体系建设，从源头上预防和治理腐败。进一步加强自身建设，弘扬新风正气，反对歪风邪气，促进社会公平正义，努力打造为民、务实、清廉政府，以良好形象感召和凝聚全市人民共创大业。

各位代表！展望未来，韶关的发展前景十分美好。让我们更加紧密地团结在以胡锦涛同志为总书记的党中央周围，在市委的正确领导下，高举中国特色社会主义伟大旗帜，以邓小平理论和“三个代表”重要思想为指导，深入贯彻落实科学发展观，解放思想，真抓实干，为建设粤北经济强市、争当全省山区科学发展排头兵而努力奋斗！
